# Minimally Invasive Tissue Sampling as an Alternative to Complete Diagnostic Autopsies in the Context of Epidemic Outbreaks and Pandemics: The Example of Coronavirus Disease 2019 (COVID-19)

**DOI:** 10.1093/cid/ciab760

**Published:** 2021-12-15

**Authors:** Quique Bassat, Rosauro Varo, Juan Carlos Hurtado, Lorena Marimon, Melania Ferrando, Mamudo R Ismail, Carla Carrilho, Fabiola Fernandes, Pedro Castro, Maria Maixenchs, Maria Teresa Rodrigo-Calvo, José Guerrero, Antonio Martínez, Marcus V G Lacerda, Inacio Mandomando, Clara Menéndez, Miguel J Martinez, Jaume Ordi, Natalia Rakislova

**Affiliations:** 1 ISGlobal, Hospital Clínic–Universitat de Barcelona, Barcelona, Spain; 2 Centro de Investigação em Saúde de Manhiça, Maputo, Mozambique; 3 ICREA, Catalan Institution for Research and Advanced Studies, Barcelona, Madrid, Spain; 4 Pediatrics Department, Hospital Sant Joan de Déu (University of Barcelona), Barcelona, Spain; 5 Consorcio de Investigación Biomédica en Red de Epidemiología y Salud Pública (CIBERESP), Spain; 6 Department of Microbiology, Hospital Clinic of Barcelona, Universitat de Barcelona, Barcelona, Spain; 7 Department of Pathology, Faculty of Medicine, Eduardo Mondlane University, Maputo, Mozambique; 8 Department of Pathology, Maputo Central Hospital, Maputo, Mozambique; 9 Medical Intensive Care Unit, Hospital Clínic, Institut D’investigacions Biomèdiques August Pi i Sunyer (IDIBAPS), University of Barcelona, Barcelona, Spain; 10 Department of Pathology, Hospital Clinic of Barcelona, Universitat de Barcelona, Barcelona, Spain; 11 Fundação de Medicina Tropical Dr Heitor Vieira Dourado, Manaus, Brazil; 12 Fundação Oswaldo Cruz, Instituto Leônidas e Maria Deane-ILMD, Manaus, Brazil; 13 Instituto Nacional de Saúde (INS), Maputo, Mozambique

**Keywords:** minimally invasive tissue sampling, MITS, postmortem, outbreak, pandemic, COVID-19

## Abstract

**Background:**

Infectious diseases’ outbreak investigation requires, by definition, conducting a thorough epidemiological assessment while simultaneously obtaining biological samples for an adequate screening of potential responsible pathogens. Complete autopsies remain the gold-standard approach for cause-of-death evaluation and characterization of emerging diseases. However, for highly transmissible infections with a significant associated lethality, such as COVID-19, complete autopsies are seldom performed due to biosafety challenges, especially in low-resource settings. Minimally invasive tissue sampling (MITS) is a validated new approach based on obtaining postmortem samples from key organs and body fluids, a procedure that does not require advanced biosafety measures or a special autopsy room.

**Methods:**

We aimed to review the use of MITS or similar procedures for outbreak investigation up to 27 March 2021 and their performance for evaluating COVID-19 deaths.

**Results:**

After a literature review, we analyzed in detail the results of 20 studies conducted at international sites, whereby 216 COVID-19–related deaths were investigated. MITS provided a general and more granular understanding of the pathophysiological changes secondary to the infection and high-quality samples where the extent and degree of severe acute respiratory syndrome coronavirus 2 (SARS-CoV-2)–related damage could be evaluated.

**Conclusions:**

MITS is a useful addition in the investigation and surveillance of infections occurring in outbreaks or epidemics. Its less invasive nature makes the tool more acceptable and feasible and reduces the risk of procedure-associated contagion, using basic biosafety measures. Standardized approaches protocolizing which samples should be collected—and under which exact biosafety measures—are necessary to facilitate and expand its use globally.

The emergence of severe acute respiratory syndrome coronavirus 2 (SARS-CoV-2) in Wuhan, China, at the end of 2019, and its subsequent international spread to reach the status of pandemic [1], has changed the way we look at global health and how the world faces collectively the arrival of an infectious pandemic. Globally, SARS-CoV-2 has caused in the last 14 months over 167 million clinical cases, and well above 3.5 million deaths globally [2]. Such figures, still on the rise despite the ongoing vaccination efforts, are demonstrative of the transformative impact and sheer magnitude borne by infectious diseases.

Substantial scientific progress in understanding coronavirus disease 2019 (COVID-19) epidemiology and transmission and in the development of new preventive (including highly effective vaccines) and therapeutic strategies has been made throughout the first year of the pandemic. The amount of scientific knowledge generated in the last few months is unprecedented, with over 100 000 scientific articles published on COVID-19 just in 2020 alone, representing approximately 4% of the world’s total research output [3]. With this extraordinary excess of COVID-19–related literature, most relevant topics, including disease burden and epidemiology, transmission, clinical characterization, prevention, treatment, and diagnosis among others, have been covered. In contrast, despite the vital role of autopsy in exploring the pathogenesis of emerging diseases, the number of postmortem studies of COVID-19–related deaths has been comparatively low, considering the high associated lethality of the disease [4–6]. The main reasons for this paucity include the declining rates of postmortem studies in general and the perceived biosafety hazards related to investigating new emerging pathogens such as COVID-19 in particular. Indeed, the biosafety risks and logistical challenges associated with the manipulation of bodies in a suspected COVID-19 death imply the need for biosafety level 3 (BSL-3) facilities [7] and negative air pressure [8], together with reinforced personal protective equipment (PPE). Admittedly, very few hospitals, even in high-income countries, can meet these safety requirements to perform full autopsies for COVID-19 cases [9]. Some guidelines, however, do not advocate for the use of a BSL-3 room or negative pressure, but instead require delaying the procedure for a minimum of 3 days after death [10]. The latter requirement may be challenging to meet due to cultural considerations, including the need for urgent burials and the potential emotional effects on the grieving families. In consequence, conducting full autopsies for COVID-19–related deaths remains challenging to perform in high-income settings, and essentially unfeasible in most low- and middle-income countries.

Minimally invasive tissue sampling (MITS), also known as postmortem biopsy or minimally invasive autopsy, could be a viable alternative to full autopsy for COVID-19 cases in those settings where autopsy is difficult to perform, is not considered a priority, or directly appears unfeasible [11]. MITS is a standardized postmortem procedure, in which tissue samples from key organs (lungs, brain, liver, heart) and body fluids (blood, cerebrospinal fluid) are obtained with core-biopsy needles without opening the body and without requiring any image-based guidance tools [12]. The MITS, as we currently know it, was initially designed [13, 14] and first implemented by our group in 2013 in Mozambique [15–18] and Brazil [19] as part of a large research study, which aimed to validate MITS against full autopsy for its use in low-resource settings. These settings often lack qualified personnel, infrastructure, and resources to perform complete autopsies. In addition, one of the critical drawbacks of full autopsies in low-resource settings is its poor acceptability, due to the marked impact on the body of the procedure. These 2 factors explain why postmortem examinations are seldom conducted in these settings, beyond forensic examinations. Due to its simplicity (it does not require fully trained pathologists, as it can be successfully performed by minimally trained individuals) [20] and its minimal impact on the body, MITS has been shown to be more feasible and acceptable, and therefore easily deployable in settings with limited resources [21]. Only a few years after the validation of the technique conducted by our team between 2013 and 2015 in all age groups, including adults [17, 19], maternal deaths [18], children [15], neonates [16], and stillbirths [16], uptake of this innovative approach to postmortem sampling has been on the rise, and is driven by the high accompanying acceptability by the populations of many countries in sub-Saharan Africa and South-East Asia [22, 23]. Indeed, MITS is currently being successfully used in sub-Saharan Africa, South Asia, and South America for different mortality research and surveillance efforts [24, 25]. Moreover, our group has recently conducted a few MITS procedures in a heavily burdened COVID-19 reference center in Spain in a basic autopsy room [26].

Historically, and perhaps as a result of the perceived fear of unacceptable risk of procedure-associated contagion, postmortem investigations, and in particular full autopsies, have been scarcely utilized for the investigation of infectious disease outbreaks. The existing exceptions include, among others, a variety of full autopsy studies conducted in outbreaks of measles [27], hand-foot-and-mouth disease [28], leptospirosis [29], H1N1 influenza A [30], and previous coronavirus outbreaks [31]. MITS has been more widely used for the study of specific infections such as human immunodeficiency virus (HIV) or tuberculosis [32–34]. Variations of MITS, such as more limited targeted postmortem biopsies of a few key organs such as the brain, liver, or lungs, have also been proposed and used in the past for the investigation of specific infectious diseases occurring in outbreaks, including Nipah virus infections in Bangladesh [35], yellow fever in Brazil [36], malaria deaths in Malawi [37], or even Ebola deaths [38], although their use in viral hemorrhagic fever outbreaks remains extremely limited. In this respect, protocolized MITS (as opposed to simpler targeted postmortem biopsies) could offer a more structured and protocolized approach that may provide highly valuable information, including histopathological and microbiological analyses of the body as a whole, and not only of targeted single organs. MITS may allow a more robust identification of the cause of death of individuals who otherwise may not be amenable to an easy investigation [39].

In this study we review the published studies that have used MITS or similar approaches in suspected or confirmed cases of COVID-19, focusing on the sampling protocols, pathological findings, and biosafety aspects. We also discuss why MITS might be an adequate approach to expand the use of postmortem studies during COVID-19 and other infectious disease outbreaks in those settings lacking resources and infrastructure to perform full autopsies.

## METHODS

We conducted a bibliographical search for original peer-reviewed articles in the electronic databases PubMed and Scopus up to 27 March 2021 using the following search terms: “Minimally Invasive Autopsy,” “Minimally Invasive Tissue Sampling,” “postmortem biopsy,” “post-mortem needle biopsy,” or “post-mortem core-needle biopsies” in combination with “COVID-19” or “SARS-CoV-2” to screen for papers on MITS-like procedures in confirmed or suspected cases of COVID-19. A total of 53 publications were initially documented. Three additional papers were identified after reviewing the references. After removing the duplicates, 49 articles remained for further screening. Subsequently, 14 articles were excluded after reviewing the abstract due to absence of original results (misclassified reviews or perspective articles). Then, we reviewed the full text and further excluded 15 papers, given that 7 of them focused on needle or punch biopsies in live patients and 8 in which the body was opened (partial or full autopsies). The methodology and laboratory results of the remaining 20 articles were reviewed in detail.

## RESULTS

### Characteristics of the Selected COVID-19 MITS Studies

Between February 2020 and March 2021, 20 journal articles reporting MITS or similar postmortem procedures in suspected or confirmed COVID-19 cases were published. Thirteen studies (65%) conducted MITS during the first 6 months of the outbreak. Three studies (15%) were conducted in the first trimester of 2020 [40–42], all of them in Wuhan (China). Sixteen of the 20 studies (80%) consisted of case series, whereas 3 were individual case reports (15%); 1 study did not report the number of cases (5%). The largest series of MITS consisted of 41 cases and was conducted in Brazil between March and June of 2020. None of the studies included a complete autopsy on the same body performed after the MITS.

### Geographical Distribution of COVID-19 MITS Studies

**Figure 1.**
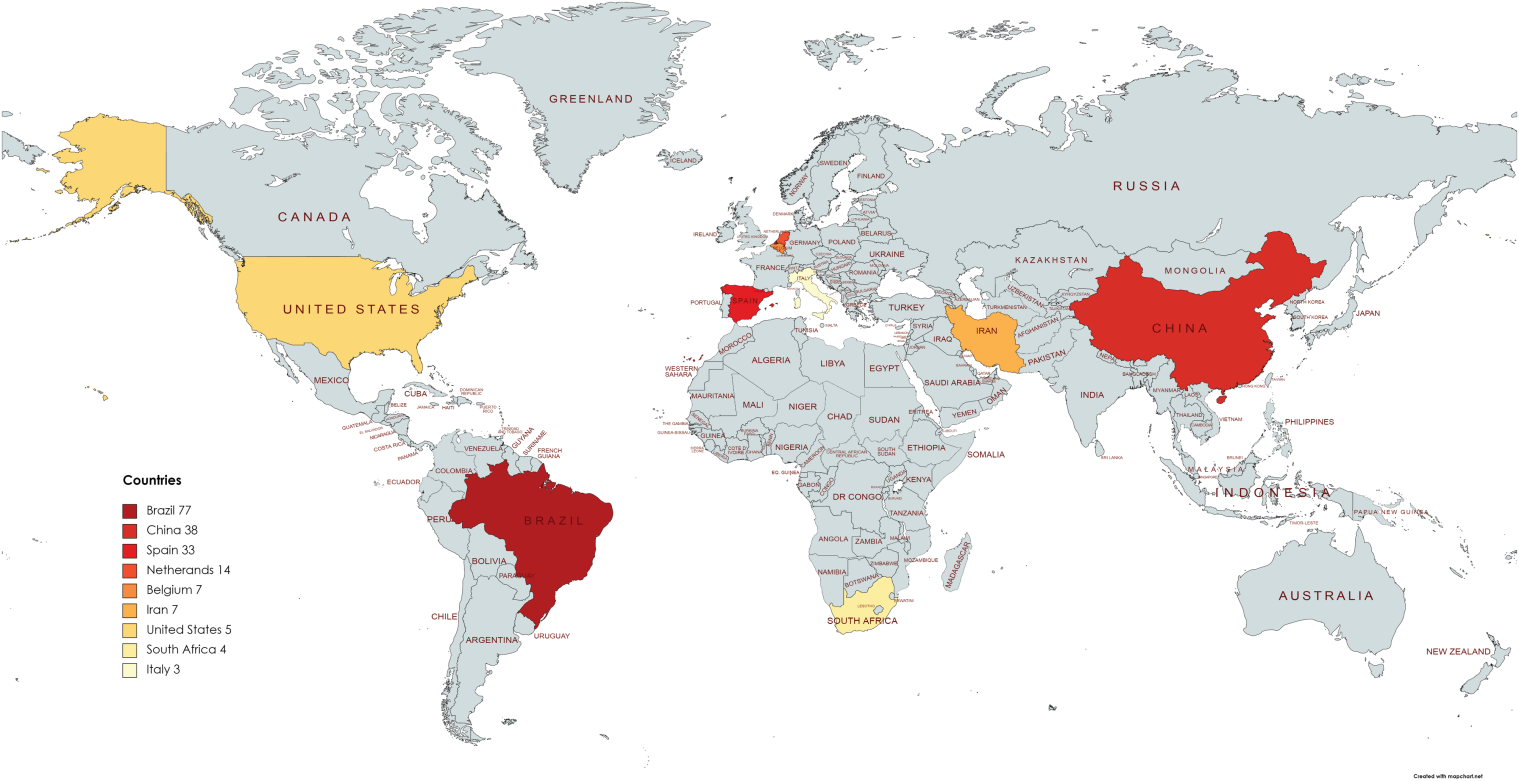
Geographic distribution of the COVID-19 MITS procedures performed in the world since the start of the pandemic. The number of MITS procedures performed in each country is shown in the map. Abbreviations: COVID-19, coronavirus disease 2019; MITS, minimally invasive tissue sampling.

A total of 216 COVID-19 MITS procedures have been reported (considering that at least 1 MITS was conducted in the study not reporting the number of MITS procedures): 214 of them performed in adults (99%) and 2 in children (1%). Eight studies (40%) have been conducted in Asia (7 in China and 1 in Iran), 6 (30%) in South America (all of them in Brazil), 4 (20%) in Europe (Spain, The Netherlands, Belgium, and Italy), 1 (5%) in North America (United States), and 1 (5%) in Africa (South Africa). No MITS studies from low-income countries were identified. No data on MITS studies were found from the Pacific (including Australia), Central America, or former Soviet Union countries. Table 1 outlines the 20 peer-reviewed postmortem studies in COVID-19 cases using an MITS approach. Figure 1 illustrates the geographic distribution of COVID-19 MITS performed in the world.

**Table 1.** Summary of Studies That Used Minimally Invasive Tissue Sampling in COVID-19 Cases During Pandemics

	Study [Reference]	Country	No. of MITS Procedures	Period of Study	Mean Age (Range), Years	Imaging Guidance	Collected Tissue Samples	RT-PCR for COVID-19 in Tissue (Yes/No)	Other Test(s) for COVID-19 in Tissue
1	Zhang et al [40]	China	1	Before March 2020	72 (N/A)	No	Lungs	No	IHC
2	Xu et al [41]	China	1	Before January 2020	50 (N/A)	No	Lungs, heart, liver	No	…
3	Tian et al [45]	China	4	Before April 2020	73 (59–81)	No	Lungs, heart, liver	Yes	…
4	Yao et al [48]	China	3	Before May 2020	70 (63–79)	No	Lung, heart, liver, kidney, spleen, bone marrow, pancreas, stomach, intestine, thyroid, and skin	Yes	IHC, EM
5	Wu et al [52]	China	10	Before June 2020	70 (39–87)	Yes (US)	Lungs	Yes	EM
6	Li et al [42]	China	30^a^	February–March 2020	69 (39–91)	Yes (US)	Lungs	No	…
7	Duarte-Neto [49]	Brazil	10	March 2020	69 (33–83)	Yes (US)	Lungs, heart, liver, kidneys, spleen, brain, skin, skeletal muscle, and testis	Yes	…
8	Dolhnikoff et al [50]	Brazil	1	Before October 2020	11 (N/A)	Yes (US)	Lungs, heart, liver, spleen, kidneys, brain, inguinal lymph node, skeletal muscle, and skin	Yes	EM
9	D’Onofrio [51]	Belgium	18	April–May 2020	80 (72–84)	Yes (CT)	Lungs, heart, liver, spleen, kidneys, abdominal fat	Yes	…
10	Flikweer et al [55]	The Netherlands	7	March–April 2020	74 (58–83)	Yes (US and CT)	Lungs	No	…
11	Matuck et al [53]	Brazil	7	Before November 2020	47 (8–74)	Yes (videoscope)	Lungs, heart, liver, kidneys, spleen, brain, skin, skeletal muscle, testis, periodontal tissue	Yes	…
12	Bruce-Brand et al [56]	South Africa	4	June–July 2020	59 (58–65)	No	Lungs	No	EM
13	Roncati et al [43]	Italy	3	Before June 2020	53 (44–67)	No	Lungs	No	EM
14	Prieto-Pérez et al [54]	Spain	33	March–April 2020	79 (53–98)	No	Lungs, bone marrow	No	IHC
15	Yang et al [46]	China	12	Before May 2020	65 (23–75)	Yes (US in 4 cases)	Testes	No	No
16	Nucci et al [47]	Brazil	NR	Before August 2020	NR	Yes (US)	Diaphragm and intercostal muscles	No	…
17	Mauaud et al [57]	Brazil	41	March–June 2020	55 (22–88)	Yes (US)	Lungs	No	…
18	Monteiro et al [58]	Brazil	28	Before January 2021	50 (0.6–83)	Yes (US)	Lungs	No	…
19	Brook et al [44]	USA	5	April–June 2020	77 (58–91)	Yes (US)	Lungs, liver, spleen, kidney, heart, testes, breast, skeletal muscle, abdominal fat	Yes	IHC, ISH sub-genomic mRNA assay
20	Beigmohammadi et al [59]	Iran	7	Before June 2020	67 (46–84)	Yes (US)	Lungs, heart, liver	No	…

Abbreviations: COVID-19, coronavirus disease 2019; CT, computed tomography; IHC, immunohistochemistry; EM, electron microscopy; ISH, in-situ hybridization; MITS, minimally invasive tissue sampling; N/A, not applicable; NR, not reported; RT-PCR, reverse transcriptase–polymerase chain reaction; US, ultrasound.

^a^10 out of 30 cases have been previously reported by Wu et al [52].

### Biosafety Rooms in COVID-19 MITS Studies

The biosafety characteristics of the rooms were reported in only 4 studies (20%). Of them, 2 studies used autopsy rooms with negative air pressure [43, 44] and 2 used negative air isolation wards [42, 45]. One additional study conducted in South Africa specified that MITS procedures were conducted in the intensive care unit, but no data on whether negative pressure was available in the room were included [10].

### Sampling in COVID-19 MITS Procedures

Thirteen studies (65%) used imaging methods to guide MITS; most of them (10/13; 77%) used ultrasound guidance. Two studies (10%) included only limited sampling of 1 specific type of tissue [46, 47], and did not include lungs or other organs key to cause-of-death attribution. In 11 studies (55%), the sampling was mainly limited to the lungs or to the lungs plus liver and/or heart. Six studies (30%) [44, 48–51] conducted a complete MITS, targeting a wide range of organs, and following the methodology proposed for MITS as a substitute of complete diagnostic autopsy [12]. Brain was sampled in 2 studies (10%), both with the guidance of ultrasound technology.

### Laboratory Analyses in the Samples Collected in COVID-19 MITS

All of the MITS studies included histological evaluation of the samples routinely stained with hematoxylin and eosin. Twelve studies (60%) performed at least 1 laboratory test to confirm the presence of COVID-19 virus in the collected samples. Of them, 4 studies (33%) applied only reverse transcriptase–polymerase chain reaction (RT-PCR), 2 (16%) used only immunohistochemistry (IHC), 2 only electron microscopy (16%), and 4 (33%) used both RT-PCR and other complementary testing (IHC or electron microscopy). Table 1 depicts the type of ancillary COVID-19 tests conducted in tissue samples in each study.

Among the 8 studies that applied SARS-CoV-2 RT-PCR in tissue samples, in 4 studies (50%) molecular analysis was conducted only in lung samples, 2 (25%) in lung and heart and/or liver, 1 (12%) in lung and bone marrow, whereas only 1 study applied it in a wide range of organs [44]. The SARS-CoV-2 genome was common in the lung samples [43, 44, 48, 52], which usually showed high viral loads. Although less frequently, viral RNA was also detected in heart [43, 58], liver [43, 58], testicles [46], and periodontal samples [50]. Immunohistochemistry for SARS-CoV-2 was consistently positive in scattered damaged type II pneumocytes [40, 48, 54]. Electron microscopy depicted viral particles in alveolar cells [52], bronchial mucosa [48], or endothelial cells [43].

### Main Pathological Findings in the COVID-19 MITS and Its Concordance With Those Reported by Full Autopsies

Among the 16 MITS studies that targeted the lungs, diffuse alveolar damage in different stages was observed in all [40–45, 48, 49, 51, 52, 54–59] except 1 study [50]. Diffuse alveolar damage was frequently associated with interstitial lymphocytic infiltrate (viral pneumonia) and superimposed bacterial pneumonia. Thrombotic lesions have been reported in 11 articles (55%), usually in the lungs (8; 73%) or kidneys (1; 9%), and occasionally in multiple sites (2; 18%). The lack of specific lesions in the lungs and the thrombotic phenomena have been consistently reported in most of the COVID-19 full autopsy series [60–65], which have not reported any additional relevant or specific COVID-19–related findings [60].

Mild inflammatory changes in the myocardium were not uncommon (3; 15%) [44, 41, 48]. Severe myocarditis has exceptionally been reported in 1 pediatric patient [50], who was diagnosed with multisystem inflammatory syndrome (MIS-C). Interestingly, viral particles were observed in this case in the cardiac cells using electron microscopy [50]. Myocarditis has also been rarely observed in COVID-19 complete autopsies in adults [63] and rarely in adolescents [66], although no viral particles were seen in cardiomyocytes in these adult patients.

Hemophagocytosis in the bone marrow has been reported in most of the MITS cases conducted in Spain [54], and has also been observed in a few complete autopsies in patients with COVID-19 [65, 67, 68]. This finding has also been reported in the spleen and the lymph nodes [65, 67, 68]. Hemophagocytosis (in spleen and lymph nodes) was also reported in a pediatric MITS case in Brazil [50]. No relevant findings were observed in 2 MITS studies targeting the brain [49, 50], although a few autopsy series have reported encephalitis [69, 70], meningitis [70, 71], or brain hemorrhage [70]. Finally, one of the MITS studies [43] reported high numbers of naked megakaryocyte nuclei in the bone marrow and lungs, which was also described in complete autopsy studies [72].

One of the MITS studies investigated periodontal tissue [53] and showed the presence of SARS-CoV-2 RNA as well as cellular vacuolization. Viral RNA was detected in the periodontal samples up to 24 days after symptoms’ onset [53]. In this regard, none of the full autopsy studies included this type of tissue in the sampling protocol. Two MITS studies explored the effect of COVID-19 on testicles in 22 cases [46, 49]. Although viral RNA was rarely detected, histological abnormalities included orchitis [49] and changes in Sertoli cells [46]; in addition, the presence of angiotensin-converting enzyme 2 (ACE2) receptor, a target of SARS-CoV-2, has been shown in germ cells [46]. Table 2 shows relevant histological findings in the COVID-19 MITS studies.

**Table 2.** Histological and Other Relevant Laboratory Findings in COVID-19 Minimally Invasive Tissue Sampling

	Study [Reference]	Lung Findings	Thrombi/ embolism	Site of Thrombi/ embolism	Other Relevant Findings
1	Zhang et al [40]	DAD	No	…	…
2	Xu [41]	DAD	No	…	Mild mononuclear infiltrates in heart and liver; lymphopenia
3	Tian [45]	DAD; BCP	No	…	…
4	Yao [48]	DAD; BCP	Yes	Kidney	Mild myocarditis; hypocellular bone marrow and spleen
5	Wu [52]	DAD; bacterial and fungal coinfection	Yes	Lungs	…
6	Li et al [42]	DAD	Yes	Lungs	…
7	Duarte-Neto [49]	DAD; BCP	Yes	Kidney, spleen, heart, skin, testis, and liver sinusoids	Pleomorphic cytopathic effects on the respiratory epithelium
8	Dolhnikoff et al [50]	Mild alveolar exudate	Yes	Lungs, kidney	Myocarditis, endocarditis, pericarditis
9	D’Onofrio [51]	DAD; bacterial and fungal coinfection	Yes	Lungs	…
10	Flikweer et al [55]	DAD; organizing pneumonia	Yes	Lungs	…
11	Matuck et al [53]	N/A	No	…	Cellular/nuclear pleomorphism and vacuolization in periodontal tissue
12	Bruce-Brand et al [56]	DAD; organizing pneumonia; BCP	Yes	Lungs	Prominent type 2 pneumocyte hyperplasia; pulmonary infarction
13	Roncati et al [43]	DAD	Yes^a^	Lungs	Clusters of megakaryocytes in the lungs
14	Prieto-Pérez et al [54]	DAD; BCP	Yes	Lungs	Hemophagocytosis in bone marrow
15	Yang et al [46]	N/A	No	…	Swelling, vacuolation, and cytoplasmic rarefaction of Sertoli cells
16	Nucci et al [47]	N/A	…	…	Muscle fibers with degeneration and atrophy
17	Mauaud et al [57]	DAD; BCP; pulmonary infarction	Yes	Lungs	…
18	Monteiro et al [58]	DAD	…	…	…
19	Brook et al [44]	DAD	…	…	Mild perivascular lymphocytic infiltrate in the heart
20	Beigmohammadi et al [59]	DAD; BCP	…	…	…

Abbreviations: BCP, bronchopneumonia; COVID-19, coronavirus disease 2019; DAD, diffuse alveolar damage; N/A, not applicable.

^a^Fibrin-rich platelet caps in small capillaries.

## DISCUSSION

### Potential Role of MITS in Future Pandemics: How to Scale Up Its Use

The investigation of infectious disease outbreaks requires, by definition, conducting a thorough epidemiological evaluation while simultaneously obtaining biological samples for an adequate screening of the potential responsible pathogens. However, for diseases with a high associated lethality there are serious concerns that postmortem evaluations may be exceedingly risky and not necessary. This is clearly exemplified by the limited studies evaluating the pathological lesions in patients who died of COVID-19. While for certain infections (eg, Ebola hemorrhagic fever) the risks of postmortem evaluation may outweigh the benefits, it would appear clear that the evaluation of tissues and bodily fluids may provide insights into the pathophysiology of new emerging infections and the relevant host response. This was the case for COVID-19, whereby postmortem examinations were initially neglected [4], but once the biosafety of the procedure was established and could be organized, these examinations started to provide extremely valuable information [73], which had not necessarily been anticipated through the accumulated clinical knowledge. Notably, MITS-like studies conducted in China during the first months of the COVID-19 outbreak [40–42] provided one of the first postmortem reports with valuable insights into COVID-19–associated tissue damage. Understanding the underlying changes in this infection at the organ level allows a more granular understanding of the pathophysiology of emerging infections such as SARS-CoV-2, and a more tailored design of preventive and therapeutic (including adjuvant treatments) interventions.

This review highlights the need for MITS studies to improve their description of biosafety precautions undertaken during MITS so as to allow other investigators to reproduce the methodology and thus enable postmortem research for similar outbreaks. It also supports the need to develop a more standardized approach (ie, a common protocol) for the specific use of MITS for outbreak investigation. In addition, the use of different analyses in the MITS samples obtained for outbreak research, particularly for novel diseases, should be upgraded. Indeed, MITS samples have proven to be suitable to perform a range of ancillary SARS-CoV-2 testing in tissues [43, 44] and thus to evaluate the viral damage throughout the body.

Our findings suggest that less invasive postmortem sampling methodologies, such as MITS, can efficiently provide relevant information that may help understanding of a novel disease where hesitancy to perform postmortem procedures may exist due to the high associated lethality of the disease. MITS, by decreasing biosafety hazards associated with more invasive procedures, appears to be an excellent tool to research outbreaks.

In this respect, our experience confirms that MITS can be of the utmost importance in the investigation of infectious disease outbreaks. As many of these outbreaks frequently start in settings where the feasibility and general acceptance of postmortem investigations remain challenging, its comparative advantages in terms of feasibility and acceptability should place MITS as the preferred investigation. The use of MITS in future pandemics may accelerate the generation of scientific knowledge around any emerging infection, thus facilitating a more evidence-based design of strategies to mitigate their harm.

## Notes

***Supplement sponsorship.*** This supplement is sponsored by MITS Surveillance Alliance Secretariat, led by RTI International, with funding from the Bill and Melinda Gates Foundation.

***Financial support.*** This work was supported by the MITS Surveillance Alliance. This work was also supported, in whole or in part, by the Bill & Melinda Gates Foundation (grant number OPP1180554). Under the grant conditions of the Foundation, a Creative Commons Attribution 4.0 Generic License has already been assigned to the Author Accepted Manuscript version that might arise from this submission. ISGlobal receives support from the Spanish Ministry of Science and Innovation through the “Centro de Excelencia Severo Ochoa 2019-2023” Program (CEX2018-000806-S), and support from the Generalitat de Catalunya through the Centres de Recerca de Catalunya Program. Centro de Investigação em Saúde de Manhiça is supported by the Government of Mozambique and the Spanish Agency for International Development (AECID).

***Potential conflicts of interest.*** The authors: No reported conflicts of interest. All authors have submitted the ICMJE Form for Disclosure of Potential Conflicts of Interest. Conflicts that the editors consider relevant to the content of the manuscript have been disclosed.
